# In vitro assessment of integrated apex locator systems: working length accuracy, operational parameters, and mechanical effects on NiTi instruments

**DOI:** 10.4317/jced.64103

**Published:** 2026-06-29

**Authors:** Roberto Barreto Osaki, Raimundo Sales de Oliveira-Neto, Gabriel Nakamura Ramos de Luca, Ricardo Gariba da Silva, Guilherme Ferreira da Silva, Bruno Carvalho de Vasconcelos, Rodrigo Ricci Vivan, Murilo Priori Alcalde, Marco Antonio Hungaro Duarte

**Affiliations:** 1 Department of Operative Dentistry, Endodontics, and Dental Materials, Bauru School of Dentistry, University of São Paulo – USP, Bauru, Brazil; 2 Department of Restorative Dentistry, Riberião Preto School of Dentistry, University of São Paulo – Riberião Preto, Brazil; 3 Department Dentistry, University Federal of Ceara, Sobral, Brazil

## Abstract

**Background:**

This study aimed to evaluate the accuracy of four hybrid endodontic motors-VDW Gold (VDW), iRoot Pro (IRP), E-Connect S (ECS), and TriAuto ZX2 (TZX)-in maintaining length, speed, and angulation during alternating and continuous rotation kinematics and the influence of these parameters on instrument fatigue.

**Materials and Methods:**

Forty-eight mandibular incisors (n=12 per group) were used to assess apex locator accuracy during glide path procedures with alternating rotation and auto apical stop (AAS) set to the apical foramen (AF: 0.0). Positioning precision was evaluated using micro-CT, while torsional resistance of instruments was tested per ISO 3630-1. Actual speed and angulation were measured using a tachometer and Arduino system. Cyclic fatigue testing was conducted with Only One File 25.08 instruments in an artificial curved canal (60°, 5 mm radius). Data were statistically analyzed with a significance level of 5%.

**Results:**

The evaluated devices exhibited similar precision (p&gt;0.05). The TZX showed the lowest number of determinations beyond the AF (p&lt;0.05). Regarding torsional testing, no significant differences were observed between groups (p&gt;0.05). The equipment showed substantial differences compared to the values displayed on the screen (p&lt;0.05). The Optimum Glide Path (OGP) motion provided significantly higher resistance to cyclic fatigue (p&lt;0.05).

**Conclusions:**

All hybrid motors were accurate in length control, with TZX being the most conservative. Kinematics did not influence torsional resistance, but significant differences were found between programmed and real parameters.

## Introduction

The introduction of highly flexible instruments made from nickel-titanium (NiTi) alloy marked a great revolution in the preparation of curved root canals (1). This advancement enabled the development of mechanized instruments for shaping curved canals, promoting more centralized preparation and reducing the occurrence of iatrogenic errors (2 - 4). Furthermore, thermal treatments applied to NiTi alloys have led to significant improvements in their mechanical properties compared to conventional NiTi, making mechanized root canal preparation even safer and more efficient (5). Reciprocating motion was introduced in endodontics as an alternative to rotary motion, offering significant benefits such as reduced cyclic and torsional stress on endodontic instruments, thereby lowering the risk of fracture (6 , 7). This approach has become a reliable technique, particularly in curved and narrow canals, ensuring safer and more predictable shaping (8). With technological advancements, various reciprocating motors have been introduced to the global market, initially by Dentsply (X-Smart Plus - Dentsply Sirona, Charlotte, NC, USA) and VDW (Silver Reciproc - VDW GmbH, Munich, Germany), operating with predefined angles (9). Subsequently, other companies launched both corded and cordless motors, allowing customization of parameters such as reciprocation direction, angulation, speed, torque, and additional features (10). The new generation of reciprocating motors may or may not be equipped with electronic apex locators, a feature that can be used either independently or during root canal instrumentation (11). Previous studies have demonstrated the accuracy of some of these devices (11 - 13). However, there remains a scarcity of research evaluating the performance of these systems, and many recently introduced devices have yet to be assessed for the precision of their apex locators. This underscores the need for further studies to validate their clinical efficacy and safety. Another critical aspect requiring evaluation in these new-generation motors is the accuracy of the actual angulation and speed parameters during instrument operation. Braambati et al. (10) demonstrated discrepancies between manufacturer-reported specifications and the actual values achieved during clinical use. Such variations may compromise both instrument safety and efficacy, potentially increasing the risk of fracture (through cyclic or torsional fatigue) and/or reducing instrumentation efficiency during endodontic procedures. The accuracy of integrated electronic apex locators in endodontic motors and the precision of their kinematic performance during root canal preparation are critical factors that warrant comprehensive evaluation. Thus, this study aimed to comparatively assess four contemporary endodontic motor systems - VDW Gold (VDW - VDW, Munich, Germany), iRoot Pro (IRP - Easy-Bassi, Minas Gerais, Brazil), E-Connect S (ECS - Mklife, Rio Grande do Sul, Brazil), and TriAuto ZX2 (TZX - J. MORITA, Kyoto, Japan) - focusing specifically on their length maintenance precision and the effects of reciprocating motion kinematics on torsional resistance during glide path procedures. Furthermore, the investigation examined the operational accuracy of speed and angulation parameters, while also evaluating potential variations in cyclic fatigue resistance of shaping instruments attributable to different motor operational characteristics. The null hypothesis postulated no significant differences among the tested motor systems regarding either measurement precision or mechanical stress generation during reciprocating instrumentation.

## Materials and Methods

**Figure 1:**
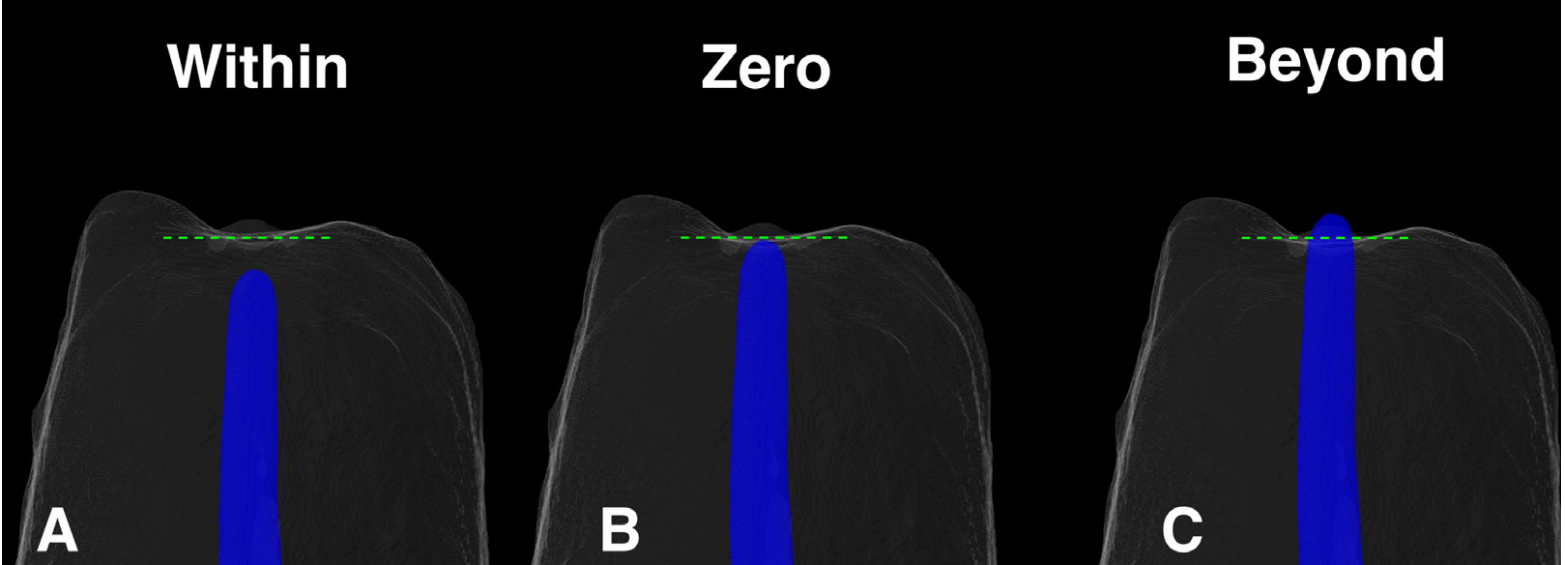
Representative images of the instrument tip and a tangent line crossing the apical foramen (AF) margins margin (A - within, B - zero and C - beyond).

The current study was approved by the Research Ethics Committee under protocol number 88828124.0.0000.5417. The sample size calculation was performed based on comparisons between experimental groups, considering a statistical design appropriate for the study objectives. A statistical power (1-) of 80% and a significance level () of 5% (two-tailed) were adopted. The sample estimation considered a minimum clinically relevant difference between groups. The calculation was performed using G*Power 3.1 software (Heinrich-Heine-Universität Düsseldorf, Düsseldorf, Germany), employing the one-way ANOVA test for the intended analysis. It was determined that n = 12 samples per group were required for the Glide Path test and torsional assay (totaling 48 samples), 10 samples for the cyclic fatigue test (totaling 40 samples), and 8 measurements per motor for angle and rotation precision analysis (totaling 32 measurements). This sample size was sufficient to detect statistically significant differences between groups while minimizing the risks of type I and II errors. - Analysis of apex locator accuracy during glide path procedures Forty-eight extracted single-rooted mandibular incisors were used, each featuring a single straight root canal, complete apical formation, and a root length of 21±1 mm. The teeth were initially visually inspected to exclude those with root dilacerations or coronal/root fractures. Subsequently, each tooth was individually radiographed in the mesiodistal direction using a digital radiography sensor (Nanopix; MK Life Medical and Dental Products, Porto Alegre, Brazil) to confirm the presence of only one canal, absence of previous endodontic treatment, and to measure radiographic tooth length. Finally, all teeth presenting a single canal were examined under a stereomicroscope (Zeiss Stemi 305; Zeiss, Oberkochen, Germany) at 40× magnification to obtain images, which were then analyzed using ImageJ software (National Institute of Mental Health, Bethesda, Maryland) to select teeth with apical foramina measuring 0.15 mm in maximum diameter. Endodontic access was prepared using #1011 and #3080 diamond burs. A size 10 K-file was then inserted into the middle third of each canal to verify root canal patency. However, no preliminary patency establishment was performed before monitored glide path procedures. The specimens were randomly allocated (random.org) into four experimental groups (n=12 per group) for evaluation of electronic apex locator accuracy in determining the 0.0 mm working length. Group assignment considered both canal length and foramen diameter measurements. This phase employed four endodontic motor systems: VDW Gold (VDW), Tri Auto ZX2 (TZX), iRoot Pro (IRP), and E-Connect S (ECS). Each tooth was mounted in a custom device featuring a lower chamber filled with freshly mixed alginate impression material (Jeltrate II; Dentsply, Petrópolis, Brazil) to simulate periodontal ligament conditions, following the methodology described by Bernardes et al. (12). The Auto Apical Stop (AAS) function was set to 0.0 mm (12 , 14). Glide path procedures were performed using an X1 15.04 instrument (MKlife) according to each motor's specific kinematics: - IRP: "OTR" mode (150° counterclockwise - 30° clockwise at 350 RPM); - VDW: "Reciproc ALL" mode (150° counterclockwise - 30° clockwise at 300 RPM); - ECS: "REC" mode (150° counterclockwise - 30° clockwise at 350 RPM); - TZX: "OGP 90" mode (90° counterclockwise - 90° clockwise / 90° counterclockwise - 120° clockwise at 300 RPM). Mechanical glide path procedures were performed using axial motions with 3-4 mm amplitude, repeated four times, followed by irrigation with 2.5 mL of 2.5% sodium hypochlorite delivered through NaviTip irrigation needles (Ultradent Products Inc, South Jordan, UT). This protocol was repeated until instrument activation was automatically interrupted upon reaching the 0.0 mm limit as indicated by the device. Upon reaching this apical limit, instruments were fixed to the teeth using cyanoacrylate adhesive (Super-Bonder; Henkel, São Paulo, Brazil). After 15 minutes of setting time, the motor was detached from the file and specimens were prepared for scanning in a SkyScan 1174v2 micro-computed tomography system (Bruker microCT, Kontich, Belgium). Teeth were scanned using predetermined parameters: 9 µm voxel size, 50 kV voltage, 800 µA current, and 0.8° rotation step at 1024×1304 resolution (14). Acquired images were reconstructed using NRecon software (v.1.6.3; Bruker-microCT) and saved in BMP format. Subsequently, the images containing instruments within root canals were analyzed using 3D Slicer software (version 4.6.0; available at https://www.slicer.org/) to visualize dentin while eliminating metal artifacts created by the alloy. For each measurement, the error was calculated as the absolute difference (in millimeters) between the instrument tip and a tangent line crossing the apical foramen (AF) margins (Fig. 1).

1

**Figure 2:**
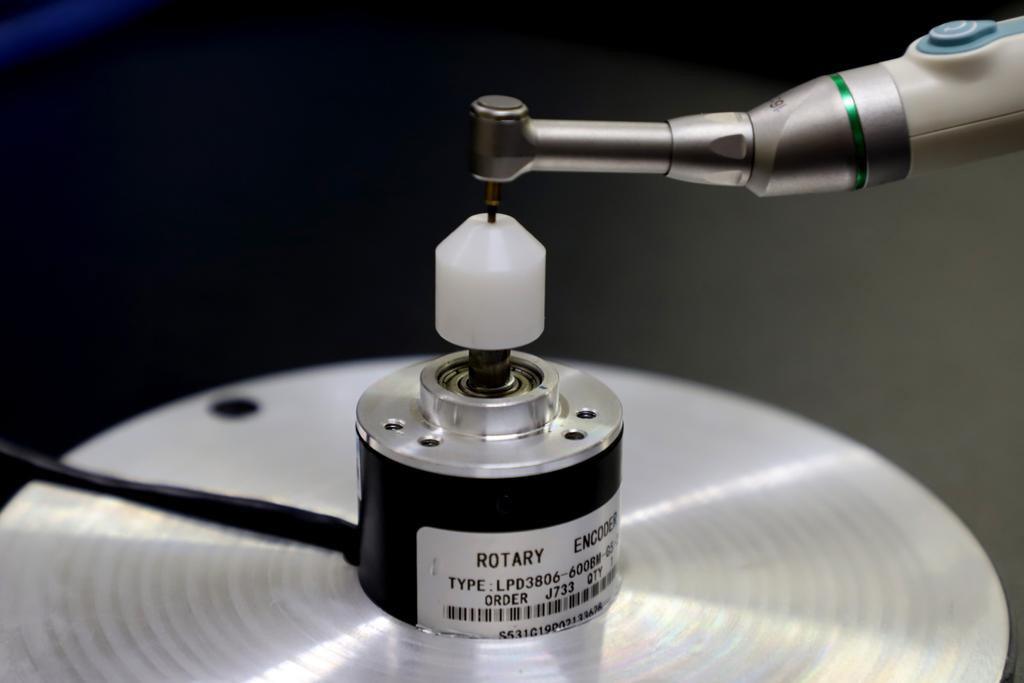
Arduino-based measurement system.

Positive and negative values were recorded when the instrument tip was detected beyond or short of the tangent line, respectively, using FIJI/ImageJ software (Fiji v.1.51n; Fiji, Madison, WI, USA). All procedures were performed by a single, previously trained operator. - Torsional Fatigue Mechanical Testing Following micro-CT scanning, the instruments were removed from the root canals and subjected to torsional fatigue testing according to ISO 3630-1 specifications, as described in previous studies (5 , 15 , 16). All tested instruments were 25 mm in length, and their handles were removed to allow proper fixation in the testing machine. A 3 mm segment of the instrument tip was secured in a chuck connected to a load cell (x N), while the opposite end was fixed in a chuck coupled to a reversible reduction motor that applied continuous counterclockwise rotation at 2 RPM. Throughout the test, torque (Ncm) and angular deflection (°) were continuously recorded. Maximum torsional load and angular deflection values were monitored and stored using specialized software (Analogica, Belo Horizonte, MG, Brazil), enabling detailed analysis of the obtained data. - Cyclic fatigue mechanical testing The cyclic fatigue test was conducted at room temperature using a custom stainless-steel apparatus designed to simulate an artificial root canal with a 60° curvature and a 5 mm radius, following established methodologies (5 , 16). A total of 40 instruments #25 (Only One file - Shenzhen Denco Medical Co., Shenzhen, China) were tested, with 10 instruments allocated per experimental group, employing the identical kinematic settings previously specified for glide path procedures. The artificial canals were lubricated with a high-viscosity specialty oil to minimize friction and instrument overheating during testing. A pre-calibrated researcher recorded time-to-fracture using a digital stopwatch, with synchronous digital videography to verify the exact moment of instrument fracture precisely. This standardized setup ensured identical laboratory conditions across all experimental groups. The number of cycles to failure (NCF) was calculated using the following formula: time to failure (in seconds) X RPM/60. - Accuracy analysis of motor-performed angulation and speed An Arduino-based measurement system was implemented to analyze the agreement between actual motor angulation and programmed settings. The system configuration consisted of resistors and connection wires interfaced with a rotary encoder mounted on a custom-designed board to measure the rotation angle of endodontic motors precisely - Fig. 2.

2

A mechanized instrument attached to the evaluated motor was secured to this assembly. The Arduino IDE platform (Arduino IDE [computer program]. Version 2.x. 2025. Available from: https://www.arduino.cc/en/software) facilitated real-time data acquisition, with recorded measurements exported into a Microsoft Office Excel 2016 spreadsheet (Microsoft Corporation, Redmond, WA, USA) for analysis. Each motor activation cycle was monitored for a standardized 60-second duration to ensure measurement consistency. A digital tachometer (MTK-3050, MetroTokyo, São Paulo, Brazil) was used to evaluate the speed accuracy of the endodontic motors. Each motor, equipped with an endodontic instrument and a resin block, was securely fixed to a stable support and connected to the digital tachometer. The motor was then activated, and speed accuracy was assessed in both continuous rotation and reciprocating kinematics. The motor was run for 2 minutes, with data from the first and last 20 seconds discarded to account for acceleration and deceleration phases. The rotational speed was recorded in revolutions per minute (RPM). Eight measurements were performed for each motor. - Statistical Analysis All experimental data were assessed for normality using the Shapiro-Wilk test. Normal distribution was confirmed for all parameters except electronic working length measurements obtained from hybrid devices, which required non-parametric analysis using Kruskal-Wallis with Dunn's post-hoc test. For normally distributed data, we employed one-way ANOVA followed by Tukey's multiple comparisons test. The significance threshold was set at 5% (=0.05), and all analyses were performed using GraphPad Prism software (version 9.3).

## Results

- Evaluation of Apex Locator Accuracy The median, minimum, and maximum values of the mean measurement error between instrument tips and the root apex during glide path procedures are presented in Table 1.

Table 1

Results showed no statistically significant differences among groups (p&gt;0.05). However, when assessing the number of specimens where measurements extended beyond the apical foramen, the TZX demonstrated the fewest occurrences, showing a statistically significant difference only when compared to the VDW group (P&lt;0.05). - Evaluation of Cyclic and Torsional Fatigue The mean values of time to fracture, number of cycles to failure (NCF), torque, and angular deflection from cyclic and torsional fatigue tests are presented in Table 2.

Table 2

Cyclic fatigue testing revealed that the OGP motion of the TZX demonstrated significantly greater NCF and time to fracture compared to other groups (p&lt;0.05). At the same time, no statistically significant differences were observed among the remaining groups (p&gt;0.05). Regarding torsional testing, instruments used in glide path procedures across all motor groups showed comparable torque and angular deflection values without statistically significant differences (p&gt;0.05). - Evaluation of Angulation and Speed Accuracy The mean and standard deviation values for the accuracy of applied angulation and speed by the motors are presented in Table 3.

Table 3

Intra-group analysis of angulation measurements revealed statistically significant differences between reference values and actual motor performance for both clockwise and counterclockwise directions in all motors, except for the TZX in clockwise rotation (p&gt;0.05). Inter-group comparisons demonstrated significant variations among motors in the discrepancy between displayed versus measured angulation values (p&lt;0.05). Notably, no statistical difference was observed between the TZX and ECS motors in clockwise rotation during inter-group directional analysis (p&gt;0.05). Regarding speed measurements, intra-group analysis showed that none of the evaluated motors-maintained agreement between displayed and measured speeds (p&lt;0.05). Inter-group comparisons revealed statistically significant differences in speed accuracy among all tested devices (p&lt;0.05).

## Discussion

This study evaluated the operational parameter accuracy of four contemporary endodontic motor systems with integrated electronic apex locators. Our investigation focused on three critical performance aspects: the precision of working length determination during glide path procedures, the consistency between displayed versus actual speed and angulation parameters, and the resultant effects on instrument fatigue resistance. The key findings revealed that all hybrid motors exhibited similar and accurate length control, with the TZX being the most conservative by showing the fewest determinations beyond the apical foramen. While kinematics did not influence torsional resistance, significant discrepancies were found between the programmed and the actual speed and angulation values for all devices. Furthermore, the TZX's OGP motion provided significantly higher resistance to cyclic fatigue compared to the other systems. The null hypothesis was therefore rejected in part. While it was supported for working length accuracy and torsional resistance, it was categorically rejected for operational precision, as significant discrepancies were found between the programmed and actual values for speed and angulation across all devices. Precise apical foramen detection is crucial to prevent over-instrumentation beyond the apical limit, thereby reducing procedural risks and potential treatment failure (11). While our results demonstrated comparable accuracy in working length determination across all tested devices, the TZX exhibited significantly fewer measurements extending beyond the apical foramen (p&lt;0.05), reinforcing its clinical safety profile during instrumentation. This study used the 0.0 mm display mark of the hybrid LEFs (i.e., the major foramen) as a reference, given its easy recognition on microCT images and consistent reproducibility (14 , 17 , 18). These findings align with Bernardes et al. (12), who emphasize the critical importance of apical precision in preventing iatrogenic errors that may compromise endodontic outcomes. The TZX demonstrated the fewest measurements beyond the apical foramen, a finding potentially attributable to its distinctive OGP kinematics (90° counterclockwise - 90° clockwise / 90° counterclockwise - 120° clockwise at 300 RPM). This motion pattern appears gentler compared to other tested systems: iRoot Pro's "OTR" function (150° CCW - 30° CW at 350 RPM), VDW Gold's "Reciproc ALL" (150° CCW - 30° CW at 300 RPM), and E-Connect S's "REC" mode (150° CCW - 30° CW at 350 RPM). The reduced apical extrusion may stem from the TZX's more balanced reciprocation angles and lower rotational stress, which could simultaneously explain its superior cyclic fatigue resistance observed in our testing. Significant discrepancies were observed between programmed and actual values for both speed and angulation across all tested devices. These findings align with Braambati et al. (10), who emphasize the critical need for strict standardization in motor control to ensure safety during curved canal preparation. Notably, torsional testing revealed no significant differences in the torsional resistance of #15/.04 Glide Path instruments (p&gt;0.05), suggesting that these speed and angulation variations did not affect their mechanical performance. This observation supports Ha et al. (19) conclusion that alternating rotation motions, regardless of specific angulation parameters, generate lower torsional stress and demonstrate comparable performance characteristics. The OGP kinematics of the TZX motor demonstrated significantly greater cycles to fracture (p&lt;0.05) compared to other tested systems. This finding substantiates that motion selection directly impacts procedural safety and predictability, particularly in canals with severe curvature (6 , 19). No significant differences were observed among other motors despite kinematic variations (p&gt;0.05), indicating that all tested devices maintain clinically acceptable safety thresholds, and the observed speed/angulation discrepancies did not affect cyclic fatigue outcomes. Sodium hypochlorite (2.5%) was used in this study because it does not adversely affect the accuracy of electronic apex locators. A previous ex vivo study specifically tested NaOCl concentrations of 0.5%, 2.5%, and 5% using two electronic apex locators (Root ZX Mini and Locapex 6). The authors found no statistical differences in working length measurements between any NaOCl concentration and visual control measurements. Within a ±0.5 mm tolerance, the Root ZX Mini achieved 88% accuracy and the Locapex 6 achieved 83% accuracy, regardless of the NaOCl concentration used (20). The present investigation utilized a static model for the assessment of cyclic and torsional fatigue resistance, a methodology well-established in the endodontic literature (5 , 16 , 21 - 23). The selection of a static testing framework is predicated on its capacity to yield fundamental data on the impact of instrument design and metallurgical properties on fatigue behavior. In dynamic models, the superimposed torsional stresses induced by contact with the walls of the artificial canal can confound fracture analysis, obscuring the distinction between failures of cyclic and torsional origin (24). Furthermore, the challenge of maintaining a purely axial motion without introducing lateral forces, which can generate aberrant stress points and compromise result validity, is a significant limitation of dynamic setups (25). Consequently, the static model was adopted to isolate the variable of interest and enhance the internal validity of the findings. It is also pertinent to note that the methodology for torsional fatigue testing remains unaffected by the choice of a dynamic or static environment (26). Another limitation of this study is that only straight, single-rooted mandibular incisors were used, even though apex locator precision is most critical in curved and complex canal anatomies. Although the use of such teeth is common in the endodontic literature (27 - 30), the results should be interpreted with caution for clinical application, given that different root canal anatomies were not tested and this is an in vitro laboratory investigation. Future studies should evaluate apex locator accuracy in teeth with varying curvatures and anatomical complexities, as well as complement these findings with clinical studies.

## Conclusions

The evaluated endodontic motors demonstrated comparable accuracy in apical length determination during glide path procedures. The TZX exhibited superior clinical performance, showing both the fewest instances of measurements extending beyond the apical foramen (p&lt;0.05 vs. VDW Gold), and significantly enhanced cyclic fatigue resistance (p&lt;0.05). While significant discrepancies between displayed versus actual speed and angulation parameters were observed across all devices (p&lt;0.05), these variations did not adversely affect either the torsional resistance of instruments or the overall cyclic fatigue performance of the motors.

## Figures and Tables

**Table 1:** Median, minimum, and maximum values (mm) of differences between instrument tip and root apex following motor-integrated apex locator-guided glide path procedures.

	Distance between instrument tip and root apex (mm)	Teeth with overextension (%)
I-Root	0,13 (-0,7- 0,53)a	6a,b
VDW	0,17 (0 – 0,41)a	8a
E-Connect S	0,02 (-0,06 – 0,76)a	5ª,b
TriAuto ZX2	-0,06 (-0,83 – 0,36)a	2b

Different lowercase letters indicate statistically significant intergroup differences (p<0.05).

**Table 2:** Mean and standard deviation values for cyclic fatigue testing of Only One File 25.08 instruments using the evaluated motors, and torsional test results of glide path X1 15.04 instruments following procedures in mandibular incisors.

	Cyclic Fatigue		Torsional Fatigue	
	Time (s)	NCF	Torque (N.cm)	Angular Deflection
I-Root	499,7 ± 19,2a	2913±112,3a	0,6±0,2a	511,4±180,8a
VDW	501,5±20,5a	2924±120a	0,6±0,1a	452,1±124a
E-Connect S	507,8±20,4a	2961±199a	0,6±0,1a	420,3±91,8a
TriAuto ZX2	1604,1±45,2b	8018±226,4b	0,6±0,1a	407,1±190,1a

Different lowercase letters indicate statistically significant intergroup differences (p<0.05).

**Table 3:** Mean and standard deviation values of motor angulation and speed accuracy: Measured angles, display reference values, angle deviation (measured vs reference), and clockwise/counterclockwise directional differences; Speed analysis including reference RPM values, digital tachometer-measured speeds, and absolute/percentage speed deviations.

	ANGLES EVALUATION						
	CW (0)		CCW (0)		Difference of the refence values		Difference Between CW and CCW
	Measured	Reference	Measured	Reference	CW	CCW	Difference
I-Root	44,5 ± 1,3a	30b	148,6±4,5a	150b	5,61±0,36A	1,3±4,5A	104,3±4,4A
VDW	38,22±2,0a	30b	159,3±3,1a	150b	-8,2±2,0B	-9,3±3,0B	121,1±3,3B
E-Connect S	64,4±5,2a	30b	192,5±5,5a	150b	-34,4±5,2C	-42,45±0,5C	128,1±5,7C
TriAuto ZX2	148,7±13,6a	90b	135,4±4,4a	90/120a	-59,9±13,5D	-30,4±15,3C,D	13,3±13,2D
SPEED EVALUATION							
	Speed Reference (RPM)		Speed Measured (RPM)		Difference Values		Diference Values (%)
I-Root	350b		192,8±13,7a		157±13,5A		44,8±3,9A
VDW	300b		163,5±10,3a		136,5±10,5B		45,4±3,4B
E-Connect S	350b		258,2±37,8a		92,1±10,5C		26,1±10,8C
TriAuto ZX2	300b		267,9±12,1a		31,1±12,1D		10,1±3,9D

Different lowercase letters indicate statistically significant intragroup differences (P < 0.05). Different capital letters indicate statistically significant intergroup differences (P < 0.05). For angulation accuracy assessments, negative values indicate measured angles exceeding display values.

## Data Availability

Data available on request from the authors.
